# A Novel Enzymatic System against Oxidative Stress in the Thermophilic Hydrogen-Oxidizing Bacterium *Hydrogenobacter thermophilus*

**DOI:** 10.1371/journal.pone.0034825

**Published:** 2012-04-02

**Authors:** Yuya Sato, Masafumi Kameya, Shinya Fushinobu, Takayoshi Wakagi, Hiroyuki Arai, Masaharu Ishii, Yasuo Igarashi

**Affiliations:** 1 Department of Biotechnology, The University of Tokyo, Bunkyo-ku, Tokyo, Japan; 2 Biotechnology Research Center and Department of Biotechnology, Toyama Prefectural University, Imizu, Toyama, Japan; Laurentian University, Canada

## Abstract

Rubrerythrin (Rbr) is a non-heme iron protein composed of two distinctive domains and functions as a peroxidase in anaerobic organisms. A novel Rbr-like protein, ferriperoxin (Fpx), was identified in *Hydrogenobacter thermophilus* and was found not to possess the rubredoxin-like domain that is present in typical Rbrs. Although this protein is widely distributed among aerobic organisms, its function remains unknown. In this study, Fpx exhibited ferredoxin:NADPH oxidoreductase (FNR)-dependent peroxidase activity and reduced both hydrogen peroxide (H_2_O_2_) and organic hydroperoxide in the presence of NADPH and FNR as electron donors. The calculated *K_m_* and *V*_max_ values of Fpx for organic hydroperoxides were comparable to that for H_2_O_2_, demonstrating a multiple reactivity of Fpx towards hydroperoxides. An *fpx* gene disruptant was unable to grow under aerobic conditions, whereas its growth profiles were comparable to those of the wild-type strain under anaerobic and microaerobic conditions, clearly indicating the indispensability of Fpx as an antioxidant of *H. thermophilus* in aerobic environments. Structural analysis suggested that domain-swapping occurs in Fpx, and this domain-swapped structure is well conserved among thermophiles, implying the importance of structural stability of domain-swapped conformation for thermal environments. In addition, Fpx was located on a deep branch of the phylogenetic tree of Rbr and Rbr-like proteins. This finding, taken together with the wide distribution of Fpx among Bacteria and Archaea, suggests that Fpx is an ancestral type of Rbr homolog that functions as an essential antioxidant and may be part of an ancestral peroxide-detoxification system.

## Introduction

Molecular oxygen (O_2_) is the most abundant oxidant existing in the earth's surface, an environment in which numerous organisms are distributed. As the aerobic respiration of O_2_ permits high energy yields, many organisms have evolved the ability to utilize O_2_. However, the utilization of O_2_ is also associated with the risk of cellular damage from reactive oxygen species (ROS) that are derived from O_2_-related metabolism. ROS have high reactivity towards numerous cellular molecules, including nucleic acids and proteins, leading to irreversible damage and even cell death. To detoxify ROS, cells are equipped with various systems, such as superoxide reductase, catalase, hydrogen peroxide reductase, and organic peroxide reductase.

Rbr is a non-heme iron homodimeric protein containing a ferritin-like diiron domain at the N-terminus and a rubredoxin (Rdx)-like domain at the C-terminus. Rbr was originally identified in the anaerobic sulfate reducing bacterium *Desulfovibrio vulgaris* and is widely distributed in anaerobic bacteria and archaea 1–6. Although Rbr has been reported to possess pyrophosphatase [7], ferroxidase [8], and superoxide dismutase (SOD) [2] activities, recent evidence suggests that it functions as a H_2_O_2_ reductase [6], [9]–[11] to protect cells against oxidative stress.

Several types of Rbr-like proteins have been identified to date [7], [12], [13]. Among them, sulerythrin (SulE), which is composed of only a ferritin-like domain, was identified in the aerobic archaea *Sulfolobus tokodaii*, however, its function remains unknown [13]. By BLAST analysis, the genes encoding this single-domain-type Rbr-like protein were found to be widely distributed among aerobic and facultative anaerobic bacteria, and archaea, whereas typical Rbr genes are distributed among anaerobes (Fig. S1). Further, it has been reported that ferritin-like diiron domain catalyzes 2-electron reduction of H_2_O_2_, and the reaction mechanism has been proposed [14]. Based on the observed distribution and reported ability to reduce H_2_O_2_, it is possible that single-domain Rbr-like proteins play a role in oxidative-stress defense systems in aerobic environments.

*Hydrogenobacter thermophilus* is a thermophilic, hydrogen-oxidizing bacterium [15] that is located at the deepest branch of the domain Bacteria based on the 16S rRNA phylogeny [16]. *H. thermophilus* is a chemolithoautotroph that assimilates carbon dioxide (CO_2_) as the sole carbon source *via* the reductive tricarboxylic acid (RTCA) cycle [17]. As several key enzymes of the RTCA cycle are highly sensitive to O_2_, the cycle commonly functions in anaerobic and microaerobic organisms [18]–[21]. However, *H. thermophilus* preferentially grows under aerobic conditions, even at O_2_ concentrations as high as 40%; thus, elucidation of the ROS detoxification system in this microorganism is of considerable interest.

In 2008, the whole-genome sequence of *H. thermophilus* was completed [22], and a gene encoding a single-domain Rbr-like protein was identified in the genome (Fig. S2). In the present study, we investigated the potential role of this Rbr-like protein as an antioxidant *in vitro* and *in vivo*. Furthermore, the structural significance and phylogeny of this protein was evaluated by comparison of the primary and tertiary structure with numerous Rbr-like proteins. Here, we show that this Rbr-like protein acts as an NADPH-FNR-dependent peroxidase and is an essential antioxidant for *H. thermophilus* in aerobic environments. We propose naming this protein ferriperoxin (Fpx) based on the observed structural and functional features.

## Results

### Heterologous expression and purification of Fpx

Recombinant Fpx was expressed in *E. coli* under control of the T7 promoter in the pET vector. The cell-free extract (CFE) of the recombinant *E. coli* strain exhibited a yellow-greenish color, suggesting that Fpx was expressed as a holoprotein, as Fpx was predicted to possess a diiron domain that is known to have absorption in the visible range (≈375 nm) [1]. A yellow-green protein was purified to homogeneity under aerobic conditions at room temperature (RT) by a combination of heat-treatment and repeated anion-exchange chromatography (Fig. S3). The N-terminal amino acid sequence of the purified protein was determined to be MKSLAGTKTL, which was identical to the deduced amino acid sequence of Fpx, indicating that the recombinant protein was derived from the *fpx* gene. The purified Fpx appeared as a single 16-kDa band on SDS-PAGE (Fig. S3A), closely corresponding to the calculated molecular mass of monomeric Fpx based on the sequence of *fpx* (15.7 kDa). To determine the subunit composition of recombinant Fpx, gel filtration was performed with purified Fpx (Fig. S3B). The molecular mass of recombinant Fpx was estimated to be 29.4 kDa, suggesting that Fpx exists as a dimeric protein, similar to typical two-domain Rbr, SulE, and rubperoxin (Rpr) isolated from other organisms [1], [12], [23], [24].

### UV-visible spectrum of Fpx

**Figure 1:**
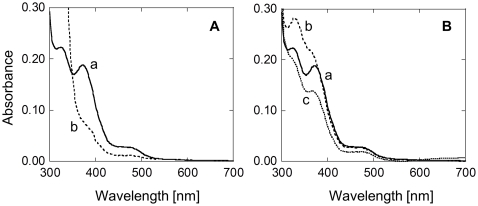
Spectrum analyses of Fpx. A. Absorption spectra of 100 µM air-oxidized (curve a) and sodium dithionite (5 mM)-reduced (curve b) Fpx in 20 mM Tris-HCl [pH 8.0] (buffer A). B. Partial reduction of Fpx by FNR/NADPH. Curve a shows the spectrum of oxidized Fpx (100 µM) in buffer A. Oxidized Fpx in buffer A was incubated with 50 µM NADPH (curve b) or with both 50 µM NADPH and 0.13 µM FNR (curve c) for 10 min at 70°C. Reduced Fpx was reoxidized by H_2_O_2_ (curve a).

Purified Fpx was yellow-green in the air-oxidized state. The UV-visible spectrum of air-oxidized Fpx (Fpx_ox_) showed a broad absorption and two peaks at 330 and 370 nm, respectively (Fig. 1A), in contrast to that of typical two-domain Rbrs, which are reported to possess peaks at 375, 500, and 580 nm [1], [25]. It has been demonstrated that the 375-nm peak corresponds to the N-terminal diiron domain, and the 500- and 580-nm peaks correspond to the C-terminal Rdx-domain in two-domain Rbrs [1]. The absence of peaks at 500 or 580 nm in the spectrum of Fpx_ox_ coincided with the absence of the Rdx domain. The characteristic peak of Fpx_ox_ at 370 nm disappeared after exposure of Fpx to the strong reductant dithionite (Fig. 1A), similar to the findings of typical two-domain Rbrs [9], [25]. This result indicates that Fpx has both oxidized and reduced states, suggesting that Fpx functions as a redox protein.

With regard to the potential physiological redox partners of Rbr homologs, two classes of candidate enzymes have been reported; one is Rdx/Rdx reductase and the second is FNR [6], [9], [25]. *H. thermophilus* possesses FNR, but homologs of Rdx or Rdx reductase have not been identified. Thus, we examined the capability of FNR to function as the redox partner of Fpx. Although no reduction of Fpx_ox_ was observed by the addition of NADPH alone to a purified protein solution (Fig. 1Bb), Fpx_ox_ was reduced when both NADPH and FNR were added, indicating that FNR was effective for mediating electron transfer from NADPH to Fpx (Fig. 1Bc). In addition, by exposure of Fpx_red_ to H_2_O_2_, the solution showed the spectrum same as oxidized Fpx, indicating that reduced Fpx was reoxidized by H_2_O_2_. This observation suggests that Fpx can function as an NADPH-FNR-dependent peroxidase.

### Enzyme assays

Rbr homologs from several organisms have been reported to possess peroxidase activity by obtaining reducing power from NAD(P)H *via* redox partners [6], [9], [25], [26]. To determine whether Fpx displays NADPH-dependent peroxidase activity, its activities toward H_2_O_2_, *t*-butyl hydroperoxide (*t*-BOOH), and cumene hydroperoxide (CMOOH) were measured using FNR as a redox partner. Fpx showed peroxidase activity when Fpx, FNR, and NADPH were present in the reaction mixture, whereas no activity was observed when any of one of three was absent (data not shown). The optimal pH for peroxidase activity of Fpx (≈pH 6.0) was lower than that of other Rbr homologs (pH 7.0 to 8.0) [6], [9], [25], [26] (Fig. S4).

**Figure 2:**
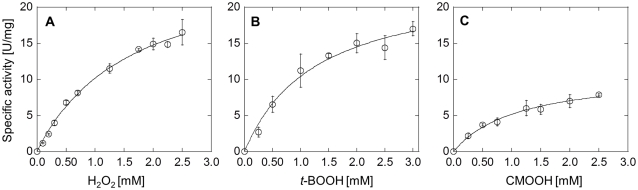
NADPH-FNR-dependent peroxidase activity of Fpx. Michaelis-Menten plots of the initial rates of removal of H_2_O_2_ (A), t-BOOH (B), or cumene hydroperoxide (CMOOH) (C) by Fpx versus the concentration of the respective peroxides. The initial rates of peroxide removal were determined directly by measuring the amount of peroxides reduced by Fpx in the presence of FNR and NADPH for 20 min at 50°C. One unit of peroxidase activity is defined as the amount of activity that can reduce 1 µmol of peroxide per min. Each value was calculated based on the measurements of triplicate samples. Error bars show the standard deviations of each triplicate values.

The initial rate of hydroperoxide consumption by Fpx was directly measured at various concentrations of H_2_O_2_, *t*-BOOH, and CMOOH in the presence of NADPH and FNR. The analysis of the resulting Michaelis-Menten plots showed that the *K_m_* values of Fpx for H_2_O_2_, *t*-BOOH, and CMOOH were 1.6±0.2, 1.3±0.3, and 1.1±0.2 mM, respectively, and the *V*_max_ values were 27±1, 24±2, and 11±1 µmol/min/mg protein, respectively (Fig. 2). The *V*_max_/*K_m_* values of Fpx for H_2_O_2_, *t*-BOOH, and CMOOH were calculated to be 17, 18, and 10 µmol/min/mg protein, respectively.

The reactivity of Fpx with superoxide produced by the xanthine/xanthine oxidase system was evaluated using a SOD Assay Kit-WST. SOD activity of Fpx was determined to be 0.10 U mg protein^−1^. This activity is very low compared to the reported values for bovine Cu, ZnSOD and *E. coli* Mn-SOD (2000-4000 U mg protein^−1^) [27], [28]. Hence, it is unlikely that Fpx is involved in superoxide anion detoxification systems.

The reactivity of Fpx with O_2_ was also analyzed. Reduced Fpx (48 µM) was slowly oxidized by dissolved O_2_ (213 µM) at an initial rate of reduction that corresponded to 0.40 nmol O_2_ min^−1^ mg protein^−1^. This calculated rate is markedly lower than to the rates of O_2_ reduction by terminal oxidases isolated from other organisms (ranging from 40–90 nmol O_2_ min^−1^ mg protein^−1^) [29], [30], indicating that Fpx is not involved in O_2_-reduction systems.

### Physiology of an *fpx* gene disruptant

**Figure 3:**
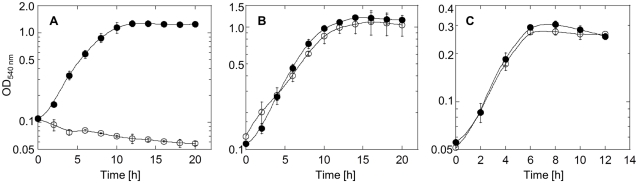
Growth curves of wild-type (WT) *H. thermophilus* and the *fpx* gene disrupt mutant (Δ*fpx*). Growth profiles of WT and Δ*fpx* were observed under aerobic (A), anaerobic (B), and microaerobic (C) conditions. The WT strain and Δ*fpx* were cultivated in inorganic medium at 70°C with shaking under aerobic (10% O_2_), anaerobic (0% O_2_, denitrification), and microaerobic (2% O_2_) conditions for 20 h. Optical density (OD) at 540 nm was monitored at 2-h intervals. Each OD was calculated based on the measurements of triplicate samples. Error bars show the standard deviations of each triplicate values. Closed and open circles denote WT and Δ*fpx*, respectively.

To investigate the *in vivo* antioxidant function of Fpx, the *fpx* gene disruptant (Δ*fpx*) was constructed by a homologous recombination method with minor alterations [31]. The disruption of the *fpx gene* was confirmed by PCR. As expected, the PCR fragment was 1.1 kb longer than that of the wild-type (WT) strain, indicating that the disruption of the *fpx* gene by insertion of the *h*ighly *t*hermostable *k*anamycin nucleotidiyltransferase (*htk*) gene gene was successful. To test the sensitivity of Δ*fpx* toward oxidative stress, the mutant and WT strains were cultured under aerobic (10% O_2_), anaerobic (0% O_2_, denitrification), and microaerobic (2% O_2_) conditions. Growth curves of the Δ*fpx* and WT strains are shown in Fig. 3. Although the growth rate of WT *H. thermophilus* was highest under aerobic conditions, Δ*fpx* was unable to grow in the presence of 10% O_2_, indicating the indispensability of Fpx for the aerobic growth of *H. thermophilus* (Fig. 3A). In contrast, the growth profiles of Δ*fpx* was comparable to that of WT under both anaerobic and microaerobic conditions.

**Figure 4:**
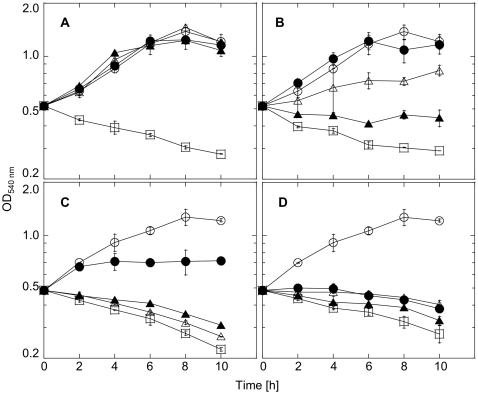
Survivals **of WT** ***H. thermophilus*** **and Δ** ***fpx*** **in the presence of ROS.** Survival profiles of WT (A, B) and Δ*fpx* (C, D) were observed under anaerobic (0% O_2_, denitrification) conditions, in the presence of paraquat (A, C) or H_2_O_2_ (B, D). Error bars show the standard deviations of each duplicate value. For panels A and C, open circle, closed circle, open triangle, closed triangle, open square denote 0, 1, 5, 10, and 100 µM paraquat. For panels B and D, open circle, closed circle, open triangle, closed triangle, open square denote 0, 10, 50, 100, and 500 µM H_2_O_2_.

To reveal whether the inhibition of the aerobic growth of Δ*fpx* resulted from the defect in the ROS detoxification system, the abilities of WT and Δ*fpx* for survival in the presence of ROS were observed (Fig. 4). WT strain was capable of growth in the presence of low concentrations of ROS (e.g. 1–10 µM paraquat and 10–50 µM H_2_O_2_). In contrast, even low concentrations of ROS showed bactericidal or inhibition effects on the mutant, strongly indicating a specific defect of the defense system against oxidative stress in Δ*fpx*. Collectively these results suggest that Fpx acts as an important antioxidant *in vivo*, and is essential for the aerobic growth of *H. thermophilus*.

### Structural modeling and phylogenetic analysis

**Figure 5:**
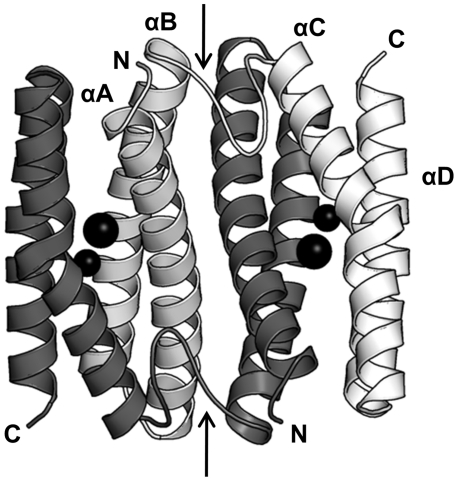
Modeled structure of Fpx. A domain-swapped homodimeric model was generated. The two polypeptide chains are shown in white and gray, and helices in the white chain are labeled. Binuclear metals are shown as black spheres. Loops connecting the helices αB and αC are indicated by arrows.

By homology modeling, the three-dimensional structure of Fpx was predicted using the crystal structure of SulE as a template (Fig. 5). The primary structure of Fpx shares 69% identity with that of SulE. The homology modeling also predicted that domain-swapping, in which a secondary or tertiary element of a monomeric protein is replaced by the same element of another protein, was predicted to occur in Fpx, similar to that reported for SulE [23].

Based on 507 amino acid sequences, the phylogenetic tree of proteins annotated as Rbr or Rbr-like proteins were constructed. The 507 sequences were divided into eight groups including out-group. Groups I, II, and III are composed of Rprs and the single-domain Rbr-like proteins, including Fpx, while typical two-domain Rbrs are distributed among groups IV to VII.

## Discussion

In this study, we demonstrated that the Rbr-like protein Fpx of *H. thermophilus* functions as a peroxidase that couples with the NADPH-FNR system. Fpx showed specific peroxidase activity toward both H_2_O_2_ and organic hydroperoxides. It was unexpected that the values of *K_m_* and *V*_max_ for *t*-BOOH and CMOOH were comparable to those for H_2_O_2_, because Rpr is reported to have almost no alkyl hydroperoxide reductase activity [12], although the activities of the other characterized Rbr homologs to date have not been reported. Thus, the multiple reactivities toward hydroperoxides appear to be a characteristic feature of Fpx. We also demonstrated by gene disruption that Fpx acts as a vital antioxidant *in vivo*. By the observation of survival profiles of Δ*fpx* in the presence of ROS, it was clearly demonstrated that the tolerance against ROS significantly decreased by defect of Fpx-system, strongly suggesting that Fpx plays a central role in the oxidative stress defense system of *H. thermophilus*. Although *H. thermophilus* utilizes the RTCA cycle, which is highly sensitive to oxidative condition, this bacterium grows preferably under aerobic conditions. Thus, Fpx may enable *H. thermophilus* to adopt such an unusual aerobic growth strategy. To our knowledge, this represents the first report to clarify the function of a single-domain Rbr-like protein as an antioxidant, and to reveal the essentiality of an Rbr-like protein for survival in aerobic environments.

The *K_m_* value of Fpx for H_2_O_2_ was greater than 40-fold higher than those of *Pyrococcus furiosus* Rbr (35 µM) and *Clostridium acetobutylicum* Rpr (<1 µM), and was similar to that of *Anabaena* sp. Rbr (2.25 mM) [6], [25], [26]. The high *K_m_* value of Fpx may imply its physiological role as a hydroperoxide scavenger, particularly in the presence of high levels of hydroperoxides. We demonstrated here that the *fpx* gene disruptant of *H. thermophilus* was unable to grow aerobically, whereas growth under anaerobic and microaerobic conditions was comparable to that of the WT strain. The observed growth profiles are consistent with the above hypothesis. The absence of an *fpx* gene in *A. aeolicus*, a microaerobic species related to *H. thermophilus*, also suggests the dispensability of Fpx for the detoxification of low amounts of ROS under microaerobic conditions.

**Figure 6:**
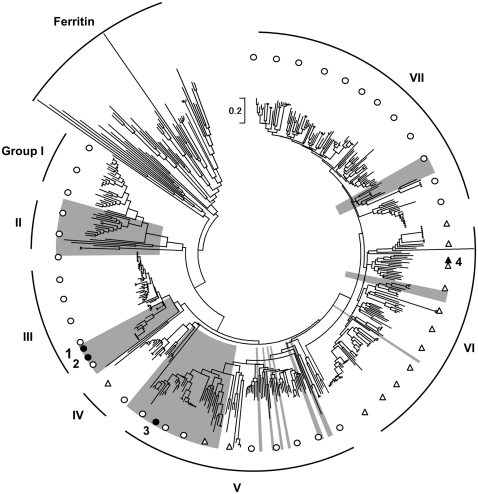
Phylogenetic tree of proteins annotated as rubrerythrin (Rbr) or Rbr-like proteins. The sequences from thermophiles are indicated by gray boxes. Circles and triangles denote domain-swapping and non-domain-swapping, respectively. Open and closed symbols denote whether each conformation was predicted by sequence alignment or experimental analyses, respectively. 1, *H. thermophilus*; 2, *S. tokodaii*; 3, *P. furiosus*; and 4, *D. vulgaris*. Group I, two-domain Rpr-type proteins from mainly Clostridia and Bacteroidia; II, single-domain Rbrs from anaerobic organisms, including Euryarchaeota; III, single-domain Fpx-SuE-type proteins from aerobic organisms, including Actinobacteria, α-, β-, γ-, and δ-proteobacteria, and Crenarchaeota; IV, two-domain Rbrs from mainly Cyanobacteria; V, two-domain Rbrs from various organisms, including Thermotogae, Clostridia, Actinobacteria, δ-proteobacteria, Euryarchaeota, and Crenarchaeota; VI, two-domain Rbrs from mainly Clostridia, Bacteroidia, and δ-proteobacteria; and VII, two-domain Rbrs from mainly Clostridia, Bacteroidia, and ε-proteobacteria. The sequences from thermophiles are indicated by gray boxes. Circles and triangles denote domain-swapping and non-domain-swapping, respectively. Open and closed symbols denote whether each conformation was predicted by sequence alignment or experimental analyses, respectively. 1, *H. thermophilus*; 2, *S. tokodaii*; 3, *P. furiosus*; and 4, *D. vulgaris*. Group I, two-domain Rpr-type proteins from mainly Clostridia and Bacteroidia; II, single-domain Rbrs from anaerobic organisms, including Euryarchaeota; III, single-domain Fpx-SuE-type proteins from aerobic organisms, including Actinobacteria, α-, β-, γ-, and δ-proteobacteria, and Crenarchaeota; IV, two-domain Rbrs from mainly Cyanobacteria; V, two-domain Rbrs from various organisms, including Thermotogae, Clostridia, Actinobacteria, δ-proteobacteria, Euryarchaeota, and Crenarchaeota; VI, two-domain Rbrs from mainly Clostridia, Bacteroidia, and δ-proteobacteria; and VII, two-domain Rbrs from mainly Clostridia, Bacteroidia, and ε-proteobacteria.

By homology modeling, domain-swapping was predicted to occur in Fpx. Moreover, domain-swapping was also observed in the crystal structure of SulE and *P. furiosus* Rbr [23], [24], [32]. All three of these homologs are harbored by thermophiles. It was reported previously that domain-swapped SulE is highly stable and is difficult to be dissociated. Hence, we speculate that a domain-swapped conformation may contribute to the structural stability of Rbr-like proteins in thermal environments. To confirm this possibility, the amino acid sequences of 507 proteins annotated as Rbr-like proteins were compared and analyzed. It was reported that the length of the loop connecting helices αB and αC of Rbr-like proteins determines whether domain-swapping occurs, with shorter loop resulting in domain swapping (Fig. S2) [23]. In the phylogenetic tree, the majority of the homologs possessed by thermophiles (groups II, III, and V) are predicted to be domain-swapped Rbr-like proteins (Fig. 6), whereas group VI including the probable non-domain-swapped homologs is composed of mainly mesophiles.

In the constructed phylogenetic tree, groups I, II, and III are composed of Rprs and the single-domain Rbr-like proteins, including Fpx. Rpr is an Rbr-like protein that was originally isolated from *C*. *acetobutylicum* [12], [26], [33] and has atypical domain composition characterized by an Rdx-like domain at the N-terminus and a ferritin-like domain at the C-terminus. Thus, although both Rpr and typical Rbr proteins possess two domains, these proteins are predicted to have different evolutionary lineages. On the other hand, as typical Rbrs are only distributed in limited areas of the phylogenetic tree (groups IV to VII), the common ancestor of Rbr-like proteins not expected to be a two-domain-type protein. In addition, as suggested by the term “evolutionary tinkering”, numerous proteins may have evolved by the recombination of small and simple proteins [34]. Considering this process of molecular evolution, a protein that has a simpler domain composition is more likely to be an ancestral protein. Therefore, typical Rbr is predicted to have arisen through the fusion of an Rdx-like domain and the ancestor of single-domain Rbr-like proteins contained in group III. In a similar manner, Rpr is predicted to have arisen from the fusion of an Rdx-like domain and the ancestor of single-domain Rbr-like proteins in group II. If these hypotheses are correct, Fpx can be considered to be an ancestral type of Rbr. As a number of small ferritin-like superfamily (FLSF) proteins have been identified and investigated recently, the phylogenetic relationship between FLSF proteins has received increased attention [35]–[37].

In conclusion, this study has demonstrated the function of Fpx as an NADPH-dependent peroxidase that is indispensable for the aerobic growth of *H. thermophilus*, and provides new insights into the distribution and enzymatic properties of Rbr-like peroxidases. Further structural and phylogenetic studies on the Rbr-like proteins are expected to uncover the specificity for both redox partners and substrates, and the molecular evolution of Rbr-like peroxidases, respectively. The genetic and transcriptional analyses of the *fpx* gene disruptant and other mutants are currently underway in our laboratory for revealing the detailed physiological role of Fpx in *H. thermophilus*.

## Materials and Methods

### Bacterial strains, media, and plasmids

*H. thermophilus* TK-6 (IAM 12695, DSMZ 6534) was cultivated at 70°C with CO_2_ as the sole carbon source in a 100-mL vial in an inorganic medium [15] for both aerobic and microaerobic conditions. For anaerobic (denitrification) conditions, the medium was supplemented with 4 g L^−1^ sodium nitrate. The head space gas in the vial was replaced with gas mixtures of H_2_:O_2_:CO_2_ (75:10:15, v/v) for aerobic conditions, H_2_:O_2_:N_2_:CO_2_ (75:2:8:15, v/v) for microaerobic conditions, and H_2_:N_2_:CO_2_ (75:10:15, v/v) for anaerobic conditions. For solid culture, liquid medium containing 10 µg L^−1^ of CuSO_4_·5H_2_O was solidified with 1.0% (w/v) of GELRITE (Wako Pure Chemical Industries, Osaka, Japan) [38]. *Escherichia coli* strains JM109 and BL21 (DE3) were used as cloning hosts for derivatives of pUC19 and pET21c (Novagen, Madison, WI), respectively. *E. coli* strains were routinely cultivated in Luria-Bertani (LB) medium at 37°C. When required, antibiotics were added to the medium at the following concentrations: 50 µg mL^−1^ kanamycin and 100 µg mL^−1^ ampicillin for *E. coli*; and 500 µg mL^−1^ kanamycin for *H. thermophilus* [31].

### Construction of plasmids for heterologous expression and homologous recombination

For heterologous protein expression, the gene encoding Fpx (*fpx*, hth_1222, GenBank Accession Number BAI69676.1) was amplified by PCR using the chromosomal DNA of *H. thermophilus* as a template with primer pairs, FpxE-F (5′-TAAGGAGCTCTACATATGAAGAGTTTAGCAGG-3′), containing an NdeI site (underlined) at the initiation codon, and FpxE-R (5′-GGCTC*AAGCTT*TAGGCTTTTAG-3′), containing an HindIII site (italics) after the stop codon. The nucleotide sequence of the obtained PCR product was confirmed on both strands. The PCR fragment was digested with NdeI and HindIII, and then ligated into NdeI-HindIII-digested pET21c. The resultant plasmid was designated pET-Fpx. For disruption of the *fpx* gene, the plasmid pFGD was constructed as follows: a 2-kb fragment carrying the *fpx* gene with KpnI sites located in the middle of the coding sequence was amplified from *H. thermophilus* chromosomal DNA by PCR with the primer pair, FpxD-F (5′-CTTTCCTGCAGTTCCGCAAAAACAG-3′), containing a PstI site (underlined) and FpxD-R (5′- GGG*GGATCC*CATAAACTACC -3′), containing a BamHI site (italics). The *htk* gene and upstream promoter [39] was amplified by PCR using primer pair, HTK-F (5′- AAAGGTACCCGTTGACGG-3′) and HTK-R (5′- AAAGGTACCGTAACCAACATGATTAACAAT-3′), with introduced KpnI sites (underlined). The amplified DNA fragment carrying the *fpx* gene was digested with PstI, KpnI, and BamHI, and then ligated with a KpnI-digested DNA fragment carrying the *htk* gene and PstI-BamHI-digested pUC19, resulting in pFGD.

### Heterologous expression and purification of recombinant Fpx

*E. coli* BL21 harboring pET-Fpx was cultivated aerobically in LB medium containing ampicillin at 37°C. When the optical density at 600 nm of the culture reached 0.6, 1 mM IPTG and 1 mM FeSO_4_ were added to the medium. After further cultivation for 3 h, cells were harvested at 10,000×*g* for 15 min, and washed once with 20 mM Tris-HCl buffer [pH 8.0]. The cells were resuspended in the same buffer, and then disrupted by sonication using an Ultrasonic disruptor (TOMY, Nerima, Tokyo, Japan) at 100 W using a 50% duty cycle for 15 min. The sonicated mixture was transferred into a vial, which was then sealed with a rubber septum and an aluminum cap, and the head space gas was replaced with argon. The vial was heat-treated at 80°C for 15 min. The heat-denatured proteins and cell debris were removed by centrifugation at 100,000×*g* for 30 min, and the resulting supernatant was used as CFE. Chromatography was performed aerobically with the ÄKTA purifier system (GE Healthcare) at RT. The purity of recombinant Fpx was monitored by the ratio of absorption at 370 nm to that at 280 nm (A_370_/A_280_) and SDS-PAGE analysis. The heat-treated extract was applied onto a Q Sepharose fast-flow column (10 mm×10 cm; GE Healthcare) equilibrated with 20 mM Tris-HCl buffer [pH 8.0] (buffer A). Proteins were eluted with a linear gradient from 0 to 0.25 M NaCl at a flow rate of 1.5 ml min^−1^. Recombinant Fpx was eluted at approximately 0.14 M NaCl. The yellow-green-colored fractions were concentrated and desalted using a VIVASPIN 6 centrifugal filter device (Sartorius Stedim Biotech GmbH, Goettingen, Germany) and loaded onto a Mono Q HR 5/5 column (1 ml bed volume; GE Healthcare) equilibrated with buffer A. Proteins were eluted with a linear gradient of 0 to 0.5 M NaCl at a flow rate of 1.0 ml min^−1^. Recombinant Fpx was eluted at approximately 0.06 M NaCl. Purified Fpx was stored at -80°C until use in a vial under anaerobic conditions by replacing the head space of cuvette with argon.

### N-terminal amino acid sequencing

Recombinant Fpx was subjected to SDS-PAGE, followed by electroblotting at 1.6 mA/cm^2^ using the HorizBlot AE-6677P Semi-dry system (Atto, Tokyo, Japan) to a Sequi-Blot^TM^ polyvinyl difluoride (PVDF) membrane (0.2 µm; Bio-Rad). The blotted protein was detected by Coomassie brilliant blue (CBB) staining. The N-terminal amino acid sequence of the detect protein was determined using a Procise 491HT protein sequencer (Applied Biosystems, Foster, CA, USA).

### Protein assay

Protein concentrations were determined using a BCA Protein Assay kit (Pierce, Rockford, IL) with bovine serum albumin as the molecular standard.

### Gel filtration

For the estimation of molecular mass, gel filtration was performed using a Superose 6 HR 10/30 column (GE Healthcare) equilibrated with buffer A containing 150 mM NaCl at flow rate of 0.5 ml min^−1^. Chromatography was performed using the ÄKTA purifier system. Gel Filtration Standards (Bio-Rad) were used as molecular markers for the calibration. All measurements of standards and samples were performed in triplicate.

### Enzyme assays

Peroxidase assays were routinely conducted anaerobically in a 200-μl reaction mixture containing 50 mM sodium phosphate [pH 6.0], 0.16 µM Fpx, 0.0037 µM FNR, 0.5 mM NADPH, and various concentrations of H_2_O_2_, *t*-BOOH, or CMOOH at 50°C for 20 min. The reaction was started by the addition of NADPH after a 1.5-min preincubation. The determination of hydroperoxide concentrations was performed according to the report of Wolf *et al*. [40], with minor modification. Following the incubation period, 10-20 µl of the reaction mixtures were added to 1.5 ml FOX1 reagent (100 µM xylenol orange, 25 mM sulfuric acid, and 250 µM ammonium iron (II) sulfate) and then incubated at RT for 40 min, after which time color development was virtually completed. The remaining amount of hydroperoxide was monitored by measuring the absorbance at 560 nm. Due to the high sensitivity of the FOX1 detection reagent in the reaction mixtures incubated at a high temperature (70°C), the peroxidase assays were performed at 50°C and sorbitol was not included in the FOX1 reagent to minimize experimental error. A calibration curve was generated in each assay. FNR was purified as previously reported [41] and was stored at -80°C until use. SOD activity was detected using a SOD Assay Kit-WST (Dojindo Molecular Technologies) at 37°C according to the manufacturer's instruction. The rate of reduction of superoxide anion is linearly related to the xanthin oxidase activity, and is inhibited by SOD activity. The SOD activity was calculated based on the inhibition rate [42]. The rate of oxidation of reduced Fpx by O_2_ was determined using O_2_-free techniques. Completely reduced Fpx was prepared according to the report of Kawasaki *et al* [12]. Briefly, excess sodium dithionite was added to a Fpx solution (100 equiv. to Fpx), which was then desalted by passage through a PD-10 gel filtration column (Amersham, Japan) and eluted with 50 mM sodium phosphate buffer [pH 6.5] under nitrogen atmospheric conditions. O_2_-saturated buffer was prepared by purging with 100% O_2_ at 37°C for 15 min and sealing the buffer in an enclosed vial. A 1/5 volume of O_2_-saturated buffer (O_2_ concentration of 1065 µM at 37°C) was added to the eluted Fpx solution (48 µM final concentration), and the enzyme reaction was then conducted under the assumed condition of 213 µM dissolved O_2_.

### Homologous recombination of *H. thermophilus*

Strain TK-6 was cultivated in a 500-mL shaking flask containing 50 ml liquid medium on a shaker at 70°C under anaerobic conditions for 24 h. When the optical density at 540 nm of the culture reached 0.6, the cells were harvested by centrifugation from 2 ml of culture, resuspended in 20 µL of 100 mM CaCl_2_ solution, and then kept on ice for 30 min. Two µg of plasmid DNA (pFGD) in TE buffer was added to the suspension, which was further incubated on ice for 1 h. After a heat shock (70°C, 10 min), the cells were added to 500 µL liquid medium and cultivated in a 5-mL vial on a shaker at 70°C under anaerobic conditions for 6 h. The suspension was then spread on an inorganic solid medium plate containing 500 µg mL^−1^ of kanamycin. The plates were placed in a desiccator and incubated under anaerobic conditions at 70°C for 7 days and gas-exchange was conducted daily. A single colony formed on a solid medium plate was inoculated into 3 ml liquid medium containing kanamycin in a test tube, which was then sealed with a butyl cap. Incubation with shaking was performed for 1–2 days until the medium became turbid. The disruption of the *fpx* gene was confirmed by determining the length of PCR-amplified DNA fragments containing the *fpx* and *htk* genes.

### Growth of *H. thermophilus* mutant under different oxic conditions

*H. thermophilus* was precultivated in 10 ml inorganic medium in a 100-mL vial under aerobic, microaerobic or anaerobic conditions. As Δ*fpx* was unable to grow under aerobic conditions, precultivation for aerobic conditions was performed anaerobically. The preculture was inoculated into 10 ml inorganic medium in a 100-mL vial, which was then incubated at 70°C with shaking under aerobic, microaerobic, and anaerobic conditions. Optical density at 540 nm was monitored at 2-h intervals. Initial optical densities at 540 nm were adjusted to 0.1 for the aerobic and anaerobic conditions, and to 0.05 for microaerobic conditions.

### Survival of *H. thermophilus* mutant in the presence of oxidative stress

*H. thermophilus* was precultivated in 10 ml inorganic medium in a 100-mL vial under anaerobic conditions. The preculture was inoculated into 3 ml inorganic medium in a 30-mL test tube, which was then incubated at 70°C with shaking under anaerobic conditions in the presence of various concentrations of paraquat or H_2_O_2_. Optical density at 540 nm was monitored at 2-h intervals. Initial optical densities at 540 nm were adjusted to 0.5 to observe both bactericidal and growth profiles. Each optical density was calculated based on the measurements of duplicate samples, except the culture of Δ*fpx* with 10 µM paraquat.

### Modeling of three-dimensional structure

Three-dimensional structure of Fpx was generated using the SWISS-MODEL automated homology modeling server (http://swissmodel.expasy.org/)

### Construction of phylogenetic tree

Based on 507 amino acid sequences obtained from the NCBI protein data-base, the phylogenetic tree of proteins annotated as Rbr or Rbr-like proteins were constructed using the maximum-likelihood method. The 54 sequences that showed markedly high homology to ferritin were used as out-groups. The branching point of ferritin group was defined as the root of the phylogenetic tree. Detailed information of the sequences used in the analysis is shown in Table S1. Although the reliability of the order of intergroup divergences between groups III, IV, and V were low, those of the other intergroup divergences were confirmed to be high (bootstrap value >70%) with other phylogenetic trees.

### Other methods

UV-visible spectra were measured on a Beckman DU 7400 spectrophotometer. The reduction and oxidation of Fpx were observed by monitoring its spectral changes under anaerobic conditions using 1-cm-pass-length anaerobic quartz cuvettes. Chemical reduction of Fpx was achieved by the addition of 5 mM sodium dithionite to a solution containing 100 µM Fpx and 20 mM Tris-HCl [pH 8.0] (buffer A) under anaerobic conditions, which were generated by replacing the head space of cuvettes with argon. Partial reduction of Fpx by FNR/NADPH was achieved by the addition of 50 µM NADPH and 0.13 µM FNR to bufferA containing 100 µM Fpx, and incubation for 10 min at 70°C.

## Supporting Information

Figure S1 **Phylogenetic tree of 1- and 2-domain types of rubrerythrin-like proteins based on amino acid sequence.** One hundred amino acid sequences were obtained by BLAST search using the amino acid sequence of *Hydrogenobacter thermophilus* Fpx (Hth). The 100 sequences and three additional amino acid sequences of Rbrs (*Desulfovibrio vulgaris*, Dvu; *Pyrococcus furiosus*, Pfu; and *Anabaena* sp. PCC7120, Ana), which have been investigated and reported to date, were used for the phylogenetic tree. The tree was constructed by using maximum-likelihood method. The group of the 1-domain type protein consists of the sequences from organisms including: 5 α-proteobacteria (α), 26 β-proteobacteria (β), 6 γ-proteobacteria (γ), 6 δ-proteobacteria (δ), 6 actinobacteria, 4 Aquificae, 1 unclassified bacteria, 10 Thermoplotei including *Sulfolobus tokodaii* (Sto) and 1 Eukaryote. This group also includes 56 aerobic, 4 microaerobic (closed circle) and 1 anaerobic (closed triangle) (and 6 unknown) organisms. The group of the 2-domain type protein consists of the sequences from 34 anaerobic organisms.
(TIF)

Figure S2 **Amino acid sequences of Fpx, SulE, and two Rbr proteins.** Residues liganded to metal centers and those conserved among all four sequences are underlined and shown in bold, respectively. Predicted secondary structure of Fpx is shown above the sequences; dotted lines and an arrow represent the α-helix and loop, respectively. Hth, *Hydrogenobacter thermophilus* Fpx; Sto, *Sulfolobus tokodaii* SulE; Pfu, *Pyrococcus furiosus* Rbr; and Dvu, *Desulfovibrio vulgaris* Rbr.
(TIF)

Figure S3 **Characterization of recombinant Fpx, a rubrerythrin-like protein from** ***H. thermophilus*** **.** A. SDS-PAGE analysis of the purified recombinant Fpx on a 13% acrylamide gel. Lane 1, 11 µg of purified Fpx, which had an apparent molecular mass of 16 kDa. B. Elution profiles of Fpx and selected molecular standards. Molecular mass of native subunit composition of Fpx was determined by gel filtration using a Superose 6 HR column equilibrated with 20 mM Tris-F|HCl and 150 mM NaCl [pH 8.0]. The profiles of the standards are as follows: thyroglobulin, 670 kDa, 12.48 ml; γ-globulin, 158 kDa, 14.24 ml; ovalbumin, 44 kDa, 16.01 ml; myoglobin, 17 kDa, 17.0 ml; and vitamin B_12_, 1.35 kDa, 19.83 ml (from top to bottom). The molecular mass of Fpx was calculated to be 29.4 kDa.
(TIF)

Figure S4 **The pH-dependent changes of peroxidase activity of Fpx.** In order to determine the optimal pH for peroxidase activity of Fpx, pH-dependency of peroxidase activity of Fpx was analyzed. Peroxidase assays were routinely carried out anaerobically in a 200-μl reaction mixture containing 0.32 µM Fpx, 0.0074 µM FNR, 0.5 mM NADPH, 100 µM hydroperoxides (H_2_O_2_ or *t*-butyl hydroperoxide (*t*-BOOH)) and 50 mM sodium phosphate [pH 6.0], at 50°C for 20 min. The reaction was started by addition of NADPH after 1.5-min preincubation. The determination of the concentrations of hydroperoxides was performed by according to the report of Wolf *et al*. with minor modification. A, the result using H_2_O_2_; B, *t*-BOOH.
(TIF)

Table S1 **The list of organisms used for the phylogenetic tree of Rbr.**
(PDF)
