# Supplementary figures and images for: A Novel Enzymatic System against Oxidative Stress in the Thermophilic Hydrogen-Oxidizing Bacterium Hydrogenobacter thermophilus
Source: PLoS One. 2012 Apr 2;7(4):e34825. doi: 10.1371/journal.pone.0034825 (PMC3317640; doi:10.1371/journal.pone.0034825)

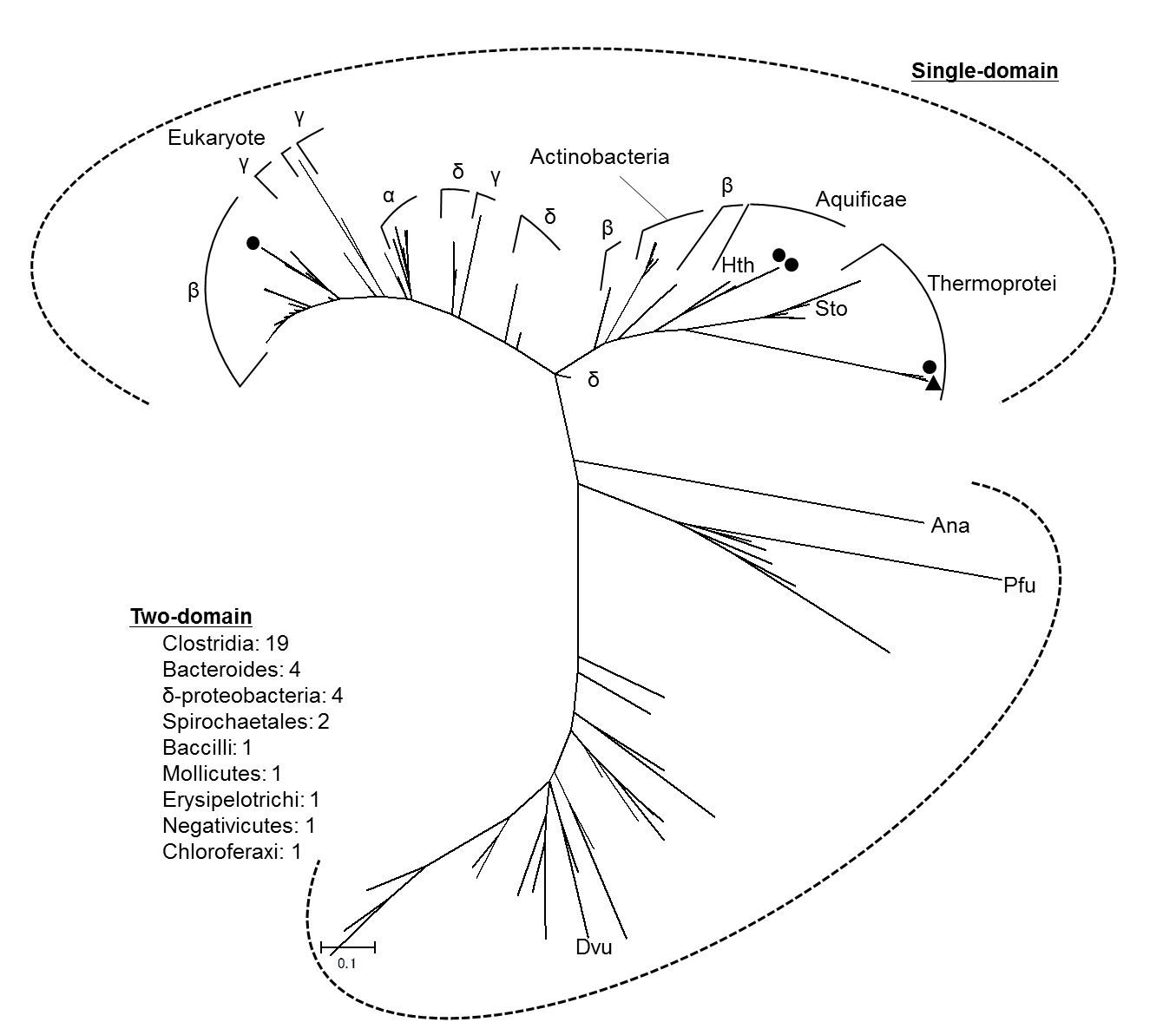

Supplement: Figure S1 — Phylogenetic tree of 1- and 2-domain types of rubrerythrin-like proteins based on amino acid sequence. One hundred amino acid sequences were obtained by BLAST search using the amino acid sequence of Hydrogenobacter thermophilus Fpx (Hth). The 100 sequences and three additional amino acid sequences of Rbrs (Desulfovibrio vulgaris, Dvu; Pyrococcus furiosus, Pfu; and Anabaena sp. PCC7120, Ana), which have been investigated and reported to date, were used for the phylogenetic tree. The tree was constructed by using maximum-likelihood method. The group of the 1-domain type protein consists of the sequences from organisms including: 5 α-proteobacteria (α), 26 β-proteobacteria (β), 6 γ-proteobacteria (γ), 6 δ-proteobacteria (δ), 6 actinobacteria, 4 Aquificae, 1 unclassified bacteria, 10 Thermoplotei including Sulfolobus tokodaii (Sto) and 1 Eukaryote. This group also includes 56 aerobic, 4 microaerobic (closed circle) and 1 anaerobic (closed triangle) (and 6 unknown) organisms. The group of the 2-domain type protein consists of the sequences from 34 anaerobic organisms. (TIF) [file pone.0034825.s001.tif]

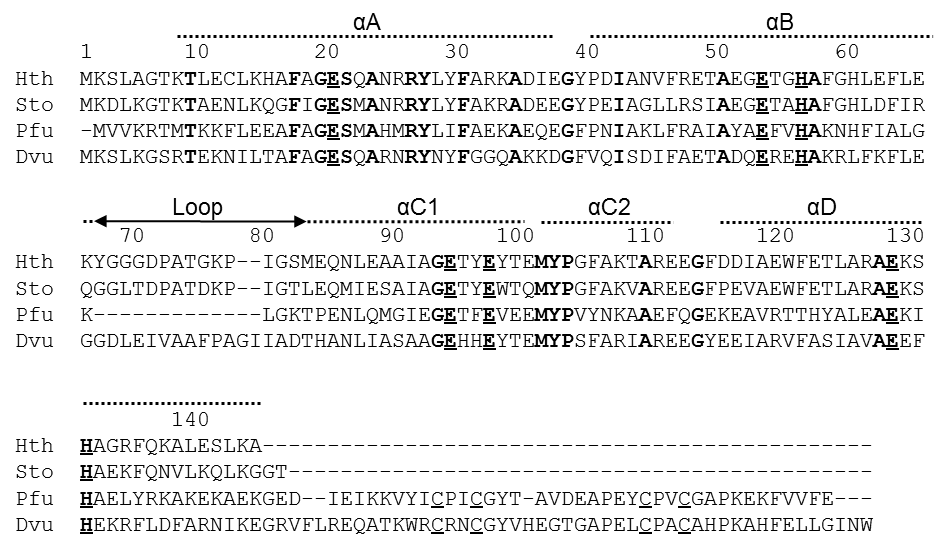

Supplement: Figure S2 — Amino acid sequences of Fpx, SulE, and two Rbr proteins. Residues liganded to metal centers and those conserved among all four sequences are underlined and shown in bold, respectively. Predicted secondary structure of Fpx is shown above the sequences; dotted lines and an arrow represent the α-helix and loop, respectively. Hth, Hydrogenobacter thermophilus Fpx; Sto, Sulfolobus tokodaii SulE; Pfu, Pyrococcus furiosus Rbr; and Dvu, Desulfovibrio vulgaris Rbr. (TIF) [file pone.0034825.s002.tif]

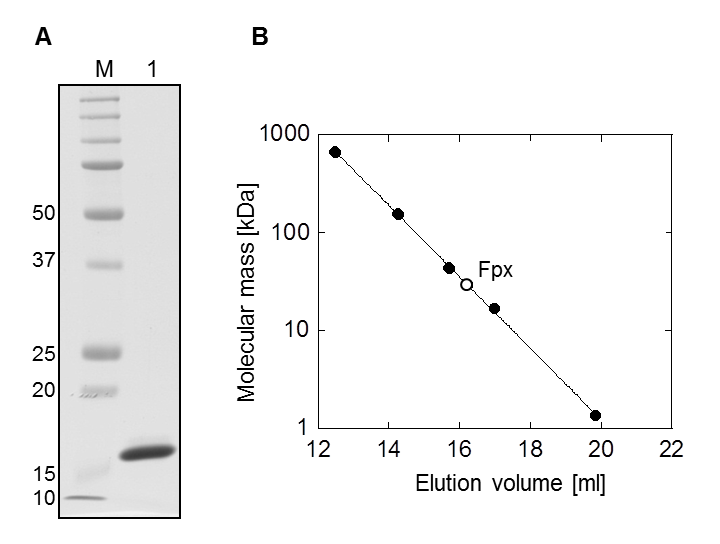

Supplement: Figure S3 — Characterization of recombinant Fpx, a rubrerythrin-like protein from H. thermophilus . A. SDS-PAGE analysis of the purified recombinant Fpx on a 13% acrylamide gel. Lane 1, 11 µg of purified Fpx, which had an apparent molecular mass of 16 kDa. B. Elution profiles of Fpx and selected molecular standards. Molecular mass of native subunit composition of Fpx was determined by gel filtration using a Superose 6 HR column equilibrated with 20 mM Tris-F|HCl and 150 mM NaCl [pH 8.0]. The profiles of the standards are as follows: thyroglobulin, 670 kDa, 12.48 ml; γ-globulin, 158 kDa, 14.24 ml; ovalbumin, 44 kDa, 16.01 ml; myoglobin, 17 kDa, 17.0 ml; and vitamin B12, 1.35 kDa, 19.83 ml (from top to bottom). The molecular mass of Fpx was calculated to be 29.4 kDa. (TIF) [file pone.0034825.s003.tif]

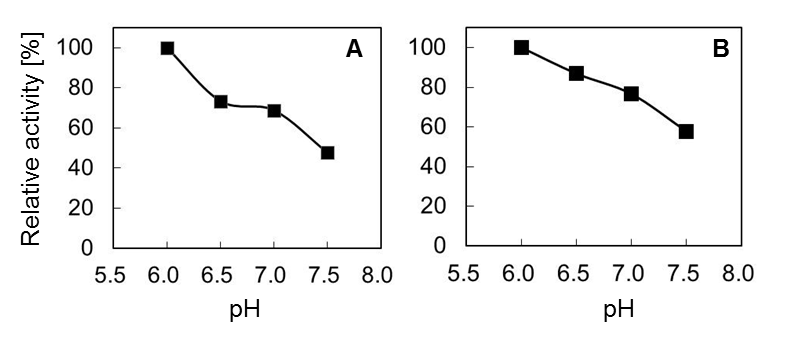

Supplement: Figure S4 — The pH-dependent changes of peroxidase activity of Fpx. In order to determine the optimal pH for peroxidase activity of Fpx, pH-dependency of peroxidase activity of Fpx was analyzed. Peroxidase assays were routinely carried out anaerobically in a 200-μl reaction mixture containing 0.32 µM Fpx, 0.0074 µM FNR, 0.5 mM NADPH, 100 µM hydroperoxides (H2O2 or t-butyl hydroperoxide (t-BOOH)) and 50 mM sodium phosphate [pH 6.0], at 50°C for 20 min. The reaction was started by addition of NADPH after 1.5-min preincubation. The determination of the concentrations of hydroperoxides was performed by according to the report of Wolf et al. with minor modification. A, the result using H2O2; B, t-BOOH. (TIF) [file pone.0034825.s004.tif]
