# Supplementary material for: A Novel Enzymatic System against Oxidative Stress in the Thermophilic Hydrogen-Oxidizing Bacterium Hydrogenobacter thermophilus
Source: PLoS One. 2012 Apr 2;7(4):e34825. doi: 10.1371/journal.pone.0034825 (PMC3317640; doi:10.1371/journal.pone.0034825)
Supplement: Table S1 — The list of organisms used for the phylogenetic tree of Rbr. (PDF) [file pone.0034825.s005.pdf]

Table S1. The list of organisms used for the phylogenetic tree of Rbr.

Accession numbers	Organisms
YP_004460534.1	*Tepidanaerobacter* sp. Re1
YP_004458226.1	*Acidianus hospitalis* W1
ZP_08424082.1	*Desulfovibrio africanus* str. Walvis Bay
YP_004123040.1	*Desulfovibrio aespoeensis* Aspo-2
YP_004059710.1	*Sulfuricurvum kujiense* DSM 16994
YP_004056882.1	*Oceanithermus profundus* DSM 14977
YP_004050876.1	*Calditerrivibrio nitroreducens* DSM 19672
YP_004043709.1	*Paludibacter propionicigenes* WB4
YP_004003988.1	*Methanothermus fervidus* DSM 2088
YP_003968682.1	*Ilyobacter polytropus* DSM 2926
YP_003913036.1	*Ferrimonas balearica* DSM 9799
YP_003892462.1	*Sulfurimonas autotrophica* DSM 16294
YP_003860023.1	*Ignisphaera aggregans* DSM 17230
YP_003827588.1	*Acetohalobium arabaticum* DSM 5501
YP_003801681.1	*Olsenella uli* DSM 7084
YP_003574679.1	*Prevotella ruminicola* 23
YP_003542677.1	*Methanohalophilus mahii* DSM 5219
YP_002830677.1	*Sulfolobus islandicus* M.14.25
YP_001960835.1	*Chlorobium phaeobacteroides* BS1
YP_001716716.1	*Candidatus Desulforudis audaxviator* MP104C
YP_001662090.1	*Thermoanaerobacter* sp. X514
YP_001665764.1	*Thermoanaerobacter pseudethanolicus* ATCC 33223
YP_001549577.1	*Methanococcus maripaludis* C6
YP_753368.1	*Syntrophomonas wolfei* subsp. *wolfei* str. Goettingen
YP_307012.1	*Methanosarcina barkeri* str. Fusaro
NP_634213.1	*Methanosarcina mazei* Go1
ZP_08420615.1	*Ruminococcaceae bacterium* D16
YP_002308840.1	*Candidatus Azobacteroides pseudotrichonymphae* genomovar. CFP2
YP_001380334.1	*Anaeromyxobacter* sp. Fw109-5
YP_001359184.1	*Sulfurovum* sp. NBC37-1
YP_903155.1	*Pelobacter propionicus* DSM 2379
YP_466298.1	*Anaeromyxobacter dehalogenans* 2CP-C
YP_002995294.1	*Thermococcus sibiricus* MM 739
YP_001887275.1	*Clostridium botulinum* B str. Eklund 17B
YP_877167.1	*Clostridium novyi* NT
YP_004441787.1	*Porphyromonas asaccharolytica* DSM 20707
YP_004440037.1	*Treponema brennaborense* DSM 12168
YP_523623.1	*Rhodoferax ferrireducens* T118
YP_462485.1	*Syntrophus aciditrophicus* SB
YP_380269.1	*Chlorobium chlorochromatii* CaD3
YP_076158.1	*Symbiobacterium thermophilum* IAM 14863
YP_004424523.1	*Pyrococcus* sp. NA2
YP_004366695.1	*Treponema succinifaciens* DSM 2489
YP_003995799.1	*Halanaerobium* sp. sapolanicus

YP_003691361.1	*Desulfurivibrio alkaliphilus* AHT2
YP_002844606.1	*Sulfolobus islandicus* M.16.27
YP_002539262.1	*Geobacter* sp. FRC-32
YP_002353537.1	*Dictyoglomus turgidum* DSM 6724
YP_001330866.1	*Methanococcus maripaludis* C7
YP_001324069.1	*Methanococcus vannielii* SB
YP_001190397.1	*Metallosphaera sedula* DSM 5348
YP_001113811.1	*Desulfotomaculum reducens* MI-1
YP_001097493.1	*Methanococcus maripaludis* C5
YP_324917.1	*Anabaena variabilis* ATCC 29413
NP_988256.1	*Methanococcus maripaludis* S2
YP_004070571.1	*Thermococcus barophilus* MP
YP_003824227.1	*Clostridium saccharolyticum* WM1
YP_002574698.1	*Campylobacter lari* RM2100
YP_001943668.1	*Chlorobium limicola* DSM 245
YP_004394873.1	*Clostridium botulinum* BKT015925
ZP_08340195.1	*Lachnospiraceae bacterium* 2 1 46FAA
YP_003346782.1	*Thermotoga naphthophila* RKU-10
YP_003128379.1	*Methanocaldococcus fervens* AG86
YP_002838987.1	*Sulfolobus islandicus* Y.G.57.14
YP_002493686.1	*Anaeromyxobacter dehalogenans* 2CP-1
YP_002467135.1	*Methanosphaerula palustris* E1-9c
YP_002364023.1	*Methylocella silvestris* BL2
YP_001918522.1	*Natranaerobius thermophilus* JW/NM-WN-LF
ZP_08324358.1	*Parasutterella excrementihominis* YIT 11859
ZP_08319394.1	*Paraprevotella xylaniphila* YIT 11841
YP_004414639.1	*Selenomonas sputigena* ATCC 35185
YP_004411557.1	*Spirochaeta coccoides* DSM 17374
YP_004410358.1	*Metallosphaera cuprina* Ar-4
YP_ 004310069.1	*Clostridium lentocellum* DSM 5427
YP_004244361.1	*Vulcanisaeta moutnovskia* 768-28
YP_004384079.1	*Methanosaeta concilii* GP-6
ZP_08301157.1	*Bacteroides fluxus* YIT 12057
ZP_08270033.1	gamma proteobacterium IMCC3088
ZP_07820717.1	*Porphyromonas asaccharolytica* PR426713P-I
ZP_07407540.1	*Clostridium difficile* QCD-32g58
YP_003852765.1	*Thermoanaerobacterium thermosaccharolyticum* DSM 571
ZP_05898666.1	*Selenomonas sputigena* ATCC 35185
ZP_05402239.1	*Clostridium difficile* QCD-23m63
YP_003023796.1	*Geobacter* sp. M21
ZP_04432267.1	*Bacillus coagulans* 36D1
YP_001952674.1	*Geobacter lovleyi* SZ
YP_002437579.1	*Desulfovibrio vulgaris* str. Miyazaki F
YP_004373534.1	*Coriobacterium glomerans* PW2
YP_004371174.1	*Desulfobacca acetoxidans* DSM 11109
YP_004370569.1	*Desulfobacca acetoxidans* DSM 11109
YP_504154.1	*Methanospirillum hungatei* JF-1

YP_503246.1	*Methanospirillum hungatei* JF-1
YP_427807.1	*Rhodospirillum rubrum* ATCC 11170
YP_551628.1	*Polaromonas* sp. JS666
YP_173147.1	*Synechococcus elongatus* PCC 6301
YP_003476329.1	*Thermoanaerobacter italicus* Ab9
YP_003436896.1	*Ferroglobus placidus* DSM 10642
YP_ 003400369.1	*Archaeoglobus profundus* DSM 5631
YP_003159947.1	*Desulfomicrobium baculatum* DSM 4028
YP_867178.1	*Magnetococcus* sp. MC-1
YP_387816.1	*Desulfovibrio desulfuricans* subsp. *desulfuricans* str. G20
YP_003990376.1	*Geobacillus* sp. Y4.1MC1
YP_004341788.1	*Archaeoglobus veneficus* SNP6
YP_004340198.1	*Hippea maritima* DSM 10411
YP_003936952.1	*Clostridium sticklandii* DSM 519
YP_003462580.1	*Dehalococcoides* sp. GT
YP_003459669.1	*Thioalkalivibrio* sp. K90mix
YP_003311280.1	*Veillonella parvula* DSM 2008
YP_002889933.1	*Thauera* sp. MZ1T
YP_002506197.1	*Clostridium cellulolyticum* H10
YP_991044.1	*Burkholderia mallei* SAVP1
NP_579012.1	*Pyrococcus furiosus* DSM 3638
ZP_07922182.1	*Pseudoramibacter alactolyticus* ATCC 23263
ZP_07547044.1	*Thermoanaerobacter wiegelii* Rt8.B1
ZP_06945956.1	*Finegoldia magna* ATCC 53516
ZP_05855897.1	*Blautia hansenii* DSM 20583
ZP_04823350.1	*Clostridium botulinum* E1 str. BoNT E Beluga
YP_003802341.1	*Spirochaeta smaragdinae* DSM 11293
YP_002862231.1	*Clostridium botulinum* Ba4 str. 657
YP_002722372.1	*Brachyspira hyodysenteriae* WA1
YP_373036.1	*Burkholderia* sp. 383
YP_438738.1	*Burkholderia thailandensis* E264
YP_004011491.1	*Rhodomicrobium vannielii* ATCC 17100
YP_002461004.1	*Desulfitobacterium hafniense* DCB-2
YP_001692903.1	*Finegoldia magna* ATCC 29328
YP_001304362.1	*Parabacteroides distasonis* ATCC 8503
YP_847360.1	*Syntrophobacter fumaroxidans* MPOB
YP_358202.1	*Pelobacter carbinolicus* DSM 2380
NP_904530.1	*Porphyromonas gingivalis* W83
YP_004026933.1	*Caldicellulosiruptor kristjanssonii* 177R1B
YP_004001733.1	*Caldicellulosiruptor owensensis* OL
YP_003993529.1	*Caldicellulosiruptor hydrothermalis* 108
YP_003473699.1	*Thermocrinis albus* DSM 14484
YP_003316624.1	*Thermanaerovibrio acidaminovorans* DSM 6589
YP_003192872.1	*Desulfotomaculum acetoxidans* DSM 771
YP_003199172.1	*Desulfohalobium retbaense* DSM 5692
YP_003180776.1	*Eggerthella lenta* DSM 2243
YP_003179149.1	*Atopobium parvulum* DSM 20469

YP_004294051.1	*Nitrosomonas* sp. AL212
YP_004291336.1	*Methanobacterium* sp. AL-21
ZP_08170188.1	*Anaerococcus hydrogenalis* ACS-025-V-Sch4
ZP_08167595.1	*Turicibacter* sp. HGF1
ZP_08130989.1	*Clostridium* sp. D5
YP_003309049.1	*Sebaldella termitidis* ATCC 33386
YP_003304374.1	*Sulfurospirillum deleyianum* DSM 6946
YP_003266639.1	*Haliangium ochraceum* DSM 14365
YP_003219215.1	*Clostridium difficile* R20291
YP_003153399.1	*Anaerococcus prevotii* DSM 20548
YP_003151240.1	*Cryptobacterium curtum* DSM 15641
ZP_04863035.1	*Clostridium botulinum* D str. 1873
NP_247719.1	*Methanocaldococcus jannaschii* DSM 2661
YP_003204968.1	*Clostridium perfringens* SM101
YP_001920386.1	*Clostridium botulinum* E3 str. Alaska E43
YP_001736950.1	*Candidatus Korarchaeum cryptofilum* OPF8
YP_001405433.1	*Candidatus Methanoregula boonei* 6A8
YP_001390684.1	*Clostridium botulinum* F str. Langeland
YP_001298450.1	*Bacteroides vulgatus* ATCC 8482
YP_001233131.1	*Geobacter uraniireducens* Rf4
YP_686166.1	uncultured methanogenic archaeon RC-I
YP_001040064.1	*Staphylothermus marinus* F1
YP_001077505.1	*Burkholderia mallei* NCTC 10247
YP_001047235.1	*Methanoculleus marisnigri* JR1
YP_921004.1	*Thermofilum pendens* Hrk 5
YP_698651.1	*Clostridium perfringens* SM101
YP_595071.1	*Lawsonia intracellularis* PHE/MN1-00
YP_447825.1	*Methanosphaera stadtmanae* DSM 3091
YP_386180.1	*Geobacter metallireducens* GS-15
YP_265823.1	*Candidatus Pelagibacter ubique* HTCC1062
YP_256851.1	*Sulfolobus acidocaldarius* DSM 639
YP_012305.1	*Desulfovibrio vulgaris* str. Hildenborough
NP_783150.1	*Clostridium tetani* E88
NP_378371.1	*Sulfolobus tokodaii* str. 7
NP_126574.1	*Pyrococcus abyssi* GE5
YP_004259291.1	*Bacteroides salanitronis* DSM 18170
YP_004267488.1	*Syntrophobotulus glycolicus* DSM 8271
YP_004252938.1	*Odoribacter splanchnicus* DSM 20712
ZP_08137106.1	*Prevotella multiformis* DSM 16608
YP_003808010.1	*Desulfarculus baarsii* DSM 2075
YP_003522931.1	*Sideroxydans lithotrophicus* ES-1
YP_002992530.1	*Desulfovibrio salexigens* DSM 2638
ZP_08091980.1	*Clostridium symbiosum* WAL-14163
ZP_08117235.1	*Thermoanaerobacterium xylanolyticum* LX-11
ZP_08115128.1	*Desulfotomaculum nigrificans* DSM 574
ZP_08111618.1	*Desulfovibrio* sp. ND132
ZP_08106438.1	*Clostridium symbiosum* WAL-14673

YP_001414893.1	*Parvibaculum lavamentivorans* DS-1
YP_002433572.1	*Desulfatibacillum alkenivorans* AK-01
YP_001791704.1	*Leptothrix cholodnii* SP-6
YP_968384.1	*Desulfovibrio vulgaris* DP4
NP_812094.1	*Bacteroides thetaiotaomicron* VPI-5482
ZP_08085101.1	*Prevotella oralis* ATCC 33269
YP_004015718.1	*Frankia* sp. EuI1c
ZP_08077252.1	*Phascolarctobacterium* sp. YIT 12067
ZP_08073645.1	*Methylocystis* sp. ATCC 49242
YP_001684045.1	*Caulobacter* sp. K31
YP_004201085.1	*Geobacter* sp. M18
ZP_08037856.1	*Treponema phagedenis* F0421
ZP_08030299.1	*Selenomonas artemidis* F0399
ZP_08028540.1	*Solobacterium moorei* F0204
YP_004196713.1	*Desulfobulbus propionicus* DSM 2032
YP_004173488.1	*Anaerolinea thermophila* UNI-1
YP_004186754.1	*Thermoanaerobacter brockii* subsp. *finnii* Ako-1
ZP_07829796.1	*Selenomonas* sp. oral taxon 137 str. F0430
YP_004167204.1	*Nitratifractor salsuginis* DSM 16511
ZP_08011949.1	*Coprobacillus* sp. 29 1
ZP_05491819.1	*Thermoanaerobacter ethanolicus* CCSD1
YP_004162400.1	*Bacteroides helcogenes* P 36-108
ZP_07994799.1	*Bacteroides* sp. 3 1 40A
ZP_07961400.1	*Prevotella salivae* DSM 15606
ZP_07959048.1	*Lachnospiraceae bacterium* 8 1 57FAA
ZP_07948139.1	*Eggerthella* sp. 1 3 56FAA
ZP_07945108.1	*Bilophila wadsworthia* 3 1 6
ZP_07938384.1	*Bacteroides* sp. 4 1 36
ZP_07936094.1	*Bacteroides eggerthii* 1 2 48FAA
ZP_07931685.1	*Anaerostipes* sp. 3 2 56FAA
ZP_06742922.1	*Bacteroides vulgatus* PC510
NP_614532.1	*Methanopyrus kandleri* AV19
YP_004092223.1	*Ethanoligenens harbinense* YUAN-3
ZP_05633354.1	*Fusobacterium ulcerans* ATCC 49185
ZP_05616993.1	*Fusobacterium* sp. 3 1 5R
ZP_07927992.1	*Fusobacterium ulcerans* ATCC 49185
ZP_07925553.1	*Fusobacterium* sp. D12
YP_004106131.1	*Ruminococcus albus* 7
YP_004112633.1	*Desulfurispirillum indicum* S5
YP_001875926.1	*Elusimicrobium minutum* Pei191
YP_001253845.1	*Clostridium botulinum* A str. ATCC 3502
YP_001227385.1	*Synechococcus* sp. RCC307
YP_001089358.1	*Clostridium difficile* 630
NP_898458.1	*Synechococcus* sp. WH 8102
YP_359426.1	*Carboxydothermus hydrogenoformans* Z-2901
ZP_07914381.1	*Fusobacterium gonidiaformans* ATCC 25563
ZP_07905665.1	*Eubacterium saburreum* DSM 3986

ZP_07893026.1	*Campylobacter upsaliensis* JV21
ZP_07882468.1	*Prevotella buccae* ATCC 33574
ZP_06249348.1	*Clostridium thermocellum* JW20
ZP_04528645.1	*Clostridium butyricum* E4 str. BoNT E BL5262
ZP_04520320.1	*Burkholderia pseudomallei* MSHR346
YP_001514333.1	*Alkaliphilus oremlandii* OhILAs
YP_001415201.1	*Xanthobacter autotrophicus* Py2
YP_001130707.1	*Prosthecochloris vibrioformis* DSM 265
YP_001115153.1	*Burkholderia vietnamiensis* G4
YP_001038185.1	*Clostridium thermocellum* ATCC 27405
YP_001025445.1	*Burkholderia mallei* NCTC 10229
YP_912017.1	*Chlorobium phaeobacteroides* DSM 266
YP_843173.1	*Methanosaeta thermophila* PT
YP_837445.1	*Burkholderia cenocepacia* HI2424
YP_741539.1	*Alkalilimnicola ehrlichii* MLHE-1
YP_624402.1	*Burkholderia cenocepacia* AU 1054
YP_567035.1	*Methanococcoides burtonii* DSM 6242
YP_393253.1	*Sulfurimonas denitrificans* DSM 1251
YP_307487.1	*Dehalococcoides* sp. CBDB1
YP_064996.1	*Desulfotalea psychrophila* LSv54
NP_971048.1	*Treponema denticola* ATCC 35405
NP_953858.1	*Geobacter sulfurreducens* PCA
NP_662214.1	*Chlorobium tepidum* TLS
NP_603352.1	*Fusobacterium nucleatum* subsp. *nucleatum* ATCC 25586
NP_562247.1	*Clostridium perfringens* str. 13
NP_275961.1	*Methanothermobacter thermautotrophicus* str. Delta H
YP_003799635.1	*Candidatus Nitrospira defluvii*
YP_482197.1	*Frankia* sp. CcI3
YP_003962098.1	*Eubacterium limosum* KIST612
YP_002016846.1	*Prosthecochloris aestuarii* DSM 271
YP_ 001995078.1	*Chloroherpeton thalassium* ATCC 35110
YP_336082.1	*Burkholderia pseudomallei* 1710b
ZP_07401718.1	*Campylobacter coli* JV20
YP_999731.1	*Campylobacter jejuni* subsp. *jejuni* 81-176
YP_421201.1	*Magnetospirillum magneticum* AMB-1
NP_350181.1	*Clostridium acetobutylicum* ATCC 824
ZP_07840009.1	*Eubacterium cellulosolvens* 6
ZP_07833370.1	*Clostridium* sp. HGF2
ZP_07827338.1	*Veillonella* sp. oral taxon 158 str. F0412
ZP_06112453.2	*Clostridium hathewayi* DSM 13479
ZP_02643906.1	*Clostridium perfringens* NCTC 8239
ZP_02693421.1	*Epulopiscium* sp. N.t. morphotype B
ZP_02622338.1	*Clostridium botulinum* C str. Eklund
ZP_06724426.1	*Bacteroides ovatus* SD CC 2a
ZP_05759155.1	*Bacteroides* sp. D2
YP_178040.1	*Campylobacter jejuni* RM1221
YP_097304.1	*Bacteroides fragilis* YCH46

YP_003850622.1	*Methanothermobacter marburgensis* str. Marburg
ZP_06258601.1	*Veillonella parvula* ATCC 17745
ZP_04055576.1	*Porphyromonas uenonis* 60-3
NP_615601.1	*Methanosarcina acetivorans* C2A
ZP_07811989.1	*Bacteroides fragilis* 3 1 12
ZP_07799452.1	*Faecalibacterium* cf. *prausnitzii* KLE1255
ZP_07757229.1	*Megasphaera micronuciformis* F0359
ZP_07738718.1	*Aminomonas paucivorans* DSM 12260
ZP_07738243.1	*Caldicellulosiruptor lactoaceticus* 6A
YP_003873714.1	*Spirochaeta thermophila* DSM 6192
ZP_07204911.1	delta proteobacterium NaphS2
ZP_06894041.1	*Clostridium difficile* NAP08
ZP_07268247.1	*Finegoldia magna* ACS-171-V-Col3
ZP_06623147.1	*Turicibacter sanguinis* PC909
YP_430722.1	*Moorella thermoacetica* ATCC 39073
YP_777422.1	*Burkholderia ambifaria* AMMD
YP_003839753.1	*Caldicellulosiruptor obsidiansis* OB47
ZP_03568334.1	*Atopobium rimae* ATCC 49626
ZP_01084886.1	*Synechococcus* sp. WH 5701
ZP_02951042.1	*Clostridium butyricum* 5521
ZP_02906630.1	*Burkholderia ambifaria* MEX-5
ZP_02190706.1	alpha proteobacterium BAL199
ZP_01906865.1	*Plesiocystis pacifica* SIR-1
ZP_01667513.1	*Thermosinus carboxydivorans* Nor1
ZP_01625227.1	marine gamma proteobacterium HTCC2080
ZP_01624162.1	Lyngbya sp. PCC 8106
ZP_01471373.1	*Synechococcus* sp. RS9916
ZP_01452211.1	*Mariprofundus ferrooxydans* PV-1
ZP_01313840.1	*Desulfuromonas acetoxidans* DSM 684
ZP_01291499.1	delta proteobacterium MLMS-1
ZP_01264435.1	*Candidatus Pelagibacter ubique* HTCC1002
ZP_01100860.1	*Campylobacter jejuni* subsp. *jejuni* 84-25
ZP_01079322.1	*Synechococcus* sp. RS9917
ZP_01072184.1	*Campylobacter jejuni* subsp. *jejuni* HB93-13
ZP_00441912.1	*Burkholderia mallei* GB8 horse 4
ZP_00371694.1	*Campylobacter upsaliensis* RM3195
ZP_00370722.1	*Campylobacter coli* RM2228
YP_001506151.1	*Frankia* sp. EAN1pec
YP_003432877.1	*Hydrogenobacter thermophilus* TK-6
YP_002017859.1	*Pelodictyon phaeoclathratiforme* BU-1
YP_001471231.1	*Thermotoga lettingae* TMO
YP_001411226.1	*Fervidobacterium nodosum* Rt17-B1
YP_001305880.1	*Thermosipho melanesiensis* BI429
YP_001244856.1	*Thermotoga petrophila* RKU-1
YP_374509.1	*Chlorobium luteolum* DSM 273
NP_228466.1	*Thermotoga maritima* MSB8
YP_003967693.1	*Ilyobacter polytropus* DSM 2926

ZP_07358698.1	*Desulfovibrio* sp. 3 1 syn3
ZP_07343228.1	*Burkholderiales bacterium* 1 1 47
ZP_07311522.1	*Streptomyces griseoflavus* Tu4000
ZP_07215720.1	*Bacteroides* sp. 20 3
ZP_07037200.1	*Peptoniphilus* sp. oral taxon 386 str. F0131
ZP_07034065.1	*Prevotella oris* C735
ZP_07001442.1	*Bacteroides* sp. D22
ZP_06854992.1	*Clostridium carboxidivorans* P7
ZP_06751181.1	*Fusobacterium* sp. 3 1 27
ZP_06646428.1	*Erysipelotrichaceae bacterium* 5 2 54FAA
ZP_06524087.1	*Fusobacterium* sp. D11
ZP_ 06423871.1	*Prevotella* sp. oral taxon 317 str. F0108
ZP_06419082.1	*Prevotella buccae* D17
ZP_06407436.1	*Prevotella melaninogenica* D18
ZP_06405604.1	*Prevotella* sp. oral taxon 299 str. F0039
ZP_06108111.1	*Brucella melitensis* bv. 3 str. Ether
ZP_06093034.1	*Bacteroides* sp. 2 1 16
ZP_05816126.1	*Fusobacterium* sp. 3 1 33
ZP_05550282.1	*Fusobacterium* sp. 3 1 36A2
ZP_05543851.1	*Parabacteroides* sp. D13
ZP_05253774.1	*Bacteroides* sp. 4 3 47FAA
ZP_05132870.1	*Clostridium* sp. 7 2 43FAA
ZP_05127634.1	gamma proteobacterium NOR5-3
ZP_05006091.1	*Streptomyces clavuligerus* ATCC 27064
ZP_04971410.1	*Fusobacterium nucleatum* subsp. *polymorphum* ATCC 10953
ZP_04946866.1	*Burkholderia dolosa* AUO158
ZP_04942159.1	*Burkholderia cenocepacia* PC184
ZP_04884073.1	*Burkholderia mallei* ATCC 10399
ZP_04880337.1	*Thermococcus* sp. AM4
ZP_04858787.1	*Fusobacterium varium* ATCC 27725
ZP_04846479.1	*Bacteroides* sp. 1 1 6
ZP_04668658.1	Clostridiales bacterium 1 7 47 FAA
ZP_04583128.1	*Helicobacter winghamensis* ATCC BAA-430
ZP_04578129.1	*Oxalobacter formigenes* OXCC13
ZP_04575589.1	*Fusobacterium* sp. 7 1
ZP_04568612.1	*Fusobacterium mortiferum* ATCC 9817
ZP_04564300.1	*Mollicutes bacterium* D7
ZP_04554784.1	*Bacteroides* sp. D4
YP_002509651.1	*Halothermothrix orenii* H 168
YP_001560902.1	*Clostridium phytofermentans* ISDg
YP_001322168.1	*Alkaliphilus metalliredigens* QYMF
YP_694597.1	*Clostridium perfringens* ATCC 13124
NP_622163.1	*Thermoanaerobacter tengcongensis* MB4
NP_485217.1	*Nostoc* (*Anabaena*) sp. PCC 7120
YP_002572382.1	*Caldicellulosiruptor bescii* DSM 6725
ZP_07670949.1	*Erysipelotrichaceae bacterium* 3 1 53
ZP_07110446.1	*Oscillatoria* sp. PCC 6506

YP_003143665.1	*Slackia heliotrinireducens* DSM 20476
YP_002952224.1	*Desulfovibrio magneticus* RS-1
YP_002941809.1	*Kosmotoga olearia* TBF 19.5.1
YP_002915915.1	*Sulfolobus islandicus* M.16.4
YP_002754596.1	*Acidobacterium capsulatum* ATCC 51196
YP_002535471.1	*Thermotoga neapolitana* DSM 4359
YP_002512913.1	*Thioalkalivibrio* sp. HL-EbGR7
YP_002379861.1	*Cyanothece* sp. PCC 7424
YP_002334465.1	*Thermosipho africanus* TCF52B
YP_002307251.1	*Thermococcus onnurineus* NA1
YP_002251357.1	*Dictyoglomus thermophilum* H-6-12
YP_002248012.1	*Thermodesulfovibrio yellowstonii* DSM 11347
YP_002135542.1	*Anaeromyxobacter* sp. K
YP_002140117.1	*Geobacter bemidjiensis* Bem
YP_001956216.1	uncultured Termite group 1 bacterium phylotype Rs-D17
YP_001928418.1	*Porphyromonas gingivalis* ATCC 33277
YP_001878662.1	*Akkermansia muciniphila* ATCC BAA-835
YP_001860284.1	*Burkholderia phymatum* STM815
YP_001810401.1	*Burkholderia ambifaria* MC40-6
YP_001777351.1	*Burkholderia cenocepacia* MC0-3
YP_001739216.1	*Thermotoga* sp. RQ2
YP_001679063.1	*Heliobacterium modesticaldum* Ice1
YP_001584887.1	*Burkholderia multivorans* ATCC 17616
YP_001568649.1	*Petrotoga mobilis* SJ95
YP_001539903.1	*Caldivirga maquilingensis* IC-167
YP_001530527.1	*Desulfococcus oleovorans* Hxd3
YP_001481589.1	*Campylobacter jejuni* subsp. jejuni 81116
YP_001405925.1	*Campylobacter hominis* ATCC BAA-381
YP_001325144.1	*Methanococcus aeolicus* Nankai-3
YP_001274306.1	*Methanobrevibacter smithii* ATCC 35061
YP_001181198.1	*Caldicellulosiruptor saccharolyticus* DSM 8903
YP_001156154.1	*Polynucleobacter necessarius* subsp. *asymbioticus* QLW-P1DMWA-1
YP_182936.1	*Thermococcus kodakarensis* KOD1
YP_003475337.1	Clostridiales genomosp. BVAB3 str. UPII9-5
ZP_07632936.1	*Clostridium cellulovorans* 743B
YP_003895809.1	*Methanoplanus petrolearius* DSM 11571
YP_003902668.1	*Vulcanisaeta distributa* DSM 14429
ZP_07579494.1	*Thermotogales bacterium* mesG1.Ag.4.2
YP_003889828.1	*Cyanothece* sp. PCC 7822
ZP_06902080.1	*Clostridium difficile* NAP07
ZP_07061479.1	*Prevotella bryantii* B14
ZP_07399442.1	*Peptoniphilus duerdenii* ATCC BAA-1640
ZP_07396923.1	*Selenomonas* sp. oral taxon 149 str. 67H29BP
YP_400666.1	*Synechococcus elongatus* PCC 7942
ZP_07321333.1	*Finegoldia magna* BVS033A4
ZP_07317176.1	*Veillonella atypica* ACS-049-V-Sch6
ZP_07334079.1	*Desulfovibrio fructosovorans* JJ

ZP_07330669.1	*Methanothermococcus okinawensis* IH1
YP_003848154.1	*Gallionella capsiferriformans* ES-2
YP_003845578.1	*Clostridium cellulovorans* 743B
YP_003357423.1	*Methanocella paludicola* SANAE
YP_003826424.1	*Thermosediminibacter oceani* DSM 16646
ZP_07269493.1	*Finegoldia magna* ACS-171-V-Col3
YP_003650478.1	*Thermosphaera aggregans* DSM 11486
YP_003786563.1	*Brachyspira pilosicoli* 95/1000
YP_003420953.1	*Sulfolobus islandicus* L.D.8.5
YP_003758808.1	*Dehalogenimonas lykanthroporepellens* BL-DC-9
YP_378185.1	*Synechococcus* sp. CC9902
YP_003727775.1	*Methanohalobium evestigatum* Z-7303
YP_412534.1	*Nitrosospira multiformis* ATCC 25196
ZP_07017088.1	*Desulfonatronospira thiodismutans* ASO3-1
ZP_06946733.1	*Finegoldia magna* ATCC 53516
YP_003707368.1	*Methanococcus voltae* A3
ZP_06760059.1	*Veillonella* sp. 3 1 44
YP_003676317.1	*Thermoanaerobacter mathranii* subsp. *mathranii* str. A3
ZP_06890064.1	*Methylosinus trichosporium* OB3b
ZP_06870983.1	*Fusobacterium nucleatum* subsp. *nucleatum* ATCC 23726
YP_003656281.1	*Arcobacter nitrofigilis* DSM 7299
YP_003640330.1	*Thermincola* sp. JR
YP_003634885.1	*Brachyspira murdochii* DSM 12563
YP_003616737.1	*Methanocaldococcus infernus* ME
YP_003246786.1	*Methanocaldococcus vulcanius* M7
ZP_06812346.1	*Geobacillus thermoglucosidasius* C56-YS93
ZP_06769268.1	*Bacteroides xylanisolvens* SD CC 1b
YP_003497178.1	*Deferribacter desulfuricans* SSM1
YP_002479482.1	*Desulfovibrio desulfuricans* subsp. *desulfuricans* str. ATCC 27774
ZP_06721218.1	*Bacteroides ovatus* SD CC 2a
YP_003554450.1	*Aminobacterium colombiense* DSM 12261
ZP_06619915.1	*Bacteroides ovatus* SD CMC 3f
ZP_05792971.1	*Butyrivibrio crossotus* DSM 2876
ZP_06604072.1	*Selenomonas noxia* ATCC 43541
YP_003249565.1	*Fibrobacter succinogenes* subsp. *succinogenes* S85
YP_003248591.1	*Fibrobacter succinogenes* subsp. *succinogenes* S85
YP_382816.1	*Synechococcus* sp. CC9605
ZP_06025864.1	*Fusobacterium periodonticum* ATCC 33693
ZP_06597599.1	*Oribacterium* sp. oral taxon 078 str. F0262
YP_003505303.1	*Denitrovibrio acetiphilus* DSM 12809
YP_003399211.1	*Acidaminococcus fermentans* DSM 20731
YP_003321998.1	*Thermobaculum terrenum* ATCC BAA-798
ZP_06347285.1	*Clostridium* sp. M62/1
YP_001409385.1	*Xanthobacter autotrophicus* Py2
ZP_06472997.1	*Frankia* symbiont of *Datisca glomerata*
ZP_06440732.1	*Anaerobaculum hydrogeniformans* ATCC BAA-1850
ZP_06425508.1	*Peptostreptococcus anaerobius* 653-L

YP_003458850.1	*Methanocaldococcus* sp. FS406-22
ZP_06416405.1	*Frankia* sp. EUN1f
ZP_05976069.1	*Methanobrevibacter smithii* DSM 2374
ZP_06392450.1	*Dethiosulfovibrio peptidovorans* DSM 11002
ZP_06370285.1	*Desulfovibrio* sp. FW1012B
ZP_06307076.1	*Cylindrospermopsis raciborskii* CS-505
ZP_06266621.1	*Pyramidobacter piscolens* W5455
ZP_05979093.1	*Subdoligranulum variabile* DSM 15176
ZP_06255253.1	*Prevotella oris* F0302
ZP_06253784.1	*Prevotella copri* DSM 18205
ZP_06245085.1	*Victivallis vadensis* ATCC BAA-548
ZP_06005113.1	*Prevotella bergensis* DSM 17361
ZP_05403918.1	*Mitsuokella multacida* DSM 20544
ZP_06143047.1	*Ruminococcus flavefaciens* FD-1
ZP_02619039.1	*Clostridium botulinum* Bf
ZP_05861411.1	*Jonquetella anthropi* E3 33 E1
ZP_05616192.1	*Faecalibacterium prausnitzii* A2-165
ZP_05916762.1	*Prevotella* sp. oral taxon 472 str. F0295
ZP_05735822.1	*Prevotella tannerae* ATCC 51259
ZP_05416994.1	*Bacteroides finegoldii* DSM 17565
ZP_05856191.1	*Prevotella veroralis* F0319
ZP_05733315.1	*Dialister invisus* DSM 15470
ZP_05473212.1	*Anaerococcus vaginalis* ATCC 51170
ZP_05440547.1	*Fusobacterium* sp. D11
ZP_05394292.1	*Clostridium carboxidivorans* P7
ZP_05385920.1	*Clostridium difficile* QCD-97b34
ZP_05323311.1	*Clostridium difficile* CIP 107932
ZP_05291555.1	*Acidithiobacillus caldus* ATCC 51756
ZP_05285227.1	*Bacteroides* sp. 2 1 7
ZP_00144835.1	*Fusobacterium nucleatum* subsp. *vincentii* ATCC 49256
ZP_04658878.1	*Selenomonas flueggei* ATCC 43531
ZP_04529185.1	*Clostridium butyricum* E4 str. BoNT E BL5262
ZP_04390080.1	*Porphyromonas endodontalis* ATCC 35406
ZP_03992648.1	*Oribacterium sinus* F0268
ZP_03989823.1	*Acidaminococcus* sp. D21
ZP_03931111.1	*Anaerococcus tetradius* ATCC 35098
ZP_03915982.1	*Anaerococcus lactolyticus* ATCC 51172
ZP_03735468.1	*Dethiobacter alkaliphilus* AHT 1
XP_001583405.1	*Trichomonas vaginalis* G3
XP 652131.1	*Entamoeba histolytica* HM-1:IMSS

The all amino acid sequences were obtained from NCBI protein data base.
